# Human Neutrophil Defensin-1, -3, and -4 Are Elevated in Nasal Aspirates from Children with Naturally Occurring Adenovirus Infection

**DOI:** 10.1155/2018/1038593

**Published:** 2018-07-31

**Authors:** V. S. Priyadharshini, F. Ramírez-Jiménez, M. Molina-Macip, C. Renteria-Rosales, J. Santiago-Cruz, Paola Zarate-Segura, E. Lara-Padilla, Luis M. Teran

**Affiliations:** ^1^Escuela Superior de Medicina del Instituto Politecnico Nacional, Plan de San Luis y Díaz Miron, Casco de Santo Tomas, 11340 Mexico City, Mexico; ^2^Instituto Nacional de Enfermedades Respiratorias “Ismael Cosío Villegas”, Calzada Tlalpan No. 4502, Sección XVI, 14080 Mexico City, Mexico; ^3^Comisión de Operación y Fomento de Actividades Académicas del IPN, Mexico City, Mexico

## Abstract

**Background:**

Adenoviruses are highly contagious pathogens which cause respiratory disease particularly in children; they may induce severe disease in infants. Human neutrophil peptides (HNPs) have been found to exhibit antiadenoviral activity. Thus, we have investigated HNPs in nasal aspirates (NAs) of children suffering from adenoviral common cold.

**Objective:**

To investigate the release of HNP-1–4 in adenovirus infection and the relationship with self-limiting upper respiratory tract infections.

**Methods:**

Nasal aspirate samples (*n*=14) were obtained from children (aged 6–12 years) infected with adenovirus between June 2012 and December 2015. Control samples were taken 4 weeks after infection when the children were asymptomatic. Levels of HNPs were measured using an enzyme-linked immunosorbent assay (ELISA).

**Results:**

There were increased levels of HNP-1, -3, and -4, but not HNP-2, in nasal aspirates (NAs) during adenovirus infections compared to healthy specimens (*p* ≤ 0.01). Moreover, there was also increase in the neutrophil count, which is a known cell source of HNPs.

**Conclusion:**

Our finding supports the involvement of HNP-1, -3, and -4 in naturally occurring cold in children infected with adenovirus. Because of their known antiviral properties, it is tempting to hypothesize that HNPs might play a protective role in adenovirus-induced respiratory disease; however, this remains to be shown.

## 1. Introduction

Viral respiratory infections in children have become a medical concern and pose financial burden around the world [1, 2]. More than 100 different viruses can infect children with adenovirus (AdV) accounting for 3–5% [3] of respiratory disease: 2–10% of bronchiolitis, 3–9% of croup, and 4–10% of pneumonia [1, 4]. While most adenoviral diseases are self-limiting, fatal illness occurs in immunocompromised patients [5] and, rarely, in healthy children and adults. In children, clinical symptoms are usually mild and include rhinorrhea, headache, fever (pharyngoconjunctival fever), and cough [6–8]. AdV are nonenveloped viruses, 70–920 nm in diameter, and its replication initiates when the viral vertices bind to the host cell surface receptors (CAR or complement protein CD46) and cell internalization occurs by endocytosis. Adenovirus has reportedly been involved in both community-acquired outbreaks [9, 10] and outbreaks of severe acute respiratory disease in military recruits in China [11, 12].

Defensins are antimicrobial peptides, effective against wide spectrum of pathogens including Gram-positive and Gram-negative bacteria, enveloped and nonenveloped viruses, and some fungi species. Humans secrete two types of defensins, namely, alpha- (6 subtypes) and beta- (31 subtypes) defensins [6, 13]. Alpha-defensins are 30–50% of total protein in neutrophils [14]. Human neutrophil peptides (HNP-1 to HNP-4) are produced by leukocytes [13], while the human defensins (HD-5 and HD-6) are secreted by intestinal paneth cells [15]. The sequences of HNP-1, -2, and -3 are almost identical. They differ only in their N-terminal amino acid, which is alanine in HNP-1 but is substituted by aspartic acid in HNP-3. However, they have different biological properties. Indeed, it was reported that HNP-4 is 100 times more potent against *E. coli* and four times more potent against both *Streptococcus faecalis* and *Candida albicans* compared with HNP-1–3 [16]. Several in vitro studies have shown that HNPs exert antiviral activity. For example, Bastian and Schäfer demonstrated that HNP-1 significantly reduced adenovirus type 5 infections of 293 cells, as compared to the normal [17]. This study investigates the presence of HNP-1–4 in nasal aspirates derived from children with naturally occurring adenovirus respiratory infections during symptomatic and asymptomatic episodes.

## 2. Methods

Between June 2012 and May 2015, 14 volunteers were recruited from 280 children registers kept in the National Institute for Respiratory Diseases (Mexico City, Mexico) from a longitudinal study on virus respiratory infection conducted in the school “Rey Tizoc,” Xochimilco, Mexico City. Inclusion criteria were as follows: (1) clinical diagnosis of nasal virus infection made by pediatrician; (2) age between 6–12 years and gender indistinct; and (3) positive immunofluorescence to adenovirus. Exclusion criteria were as follows: (1) presence of clinical symptoms that suggested bacterial infection; (2) treatment using antibiotic medication, antihistamines, nonsteroidal anti-inflammatory drugs, nasal/systemic corticosteroids, or a nasal/oral decongestant within 2 weeks of the beginning of clinical symptoms; and (3) children who had coinfection with other viruses detected by immunofluorescence assay [17]. Parents and children gave their written and verbal consent. The subjective respiratory symptoms of children were assessed using a 4-point scale: 0 = absent, 1 = mild, 2 = moderate, and 3 = severe. Upper respiratory tract symptoms were the runny nose, sneezing, blocked nose, sore throat, hoarse voice, headaches, aches or pains elsewhere, and chillness, fever, or shivers. Lower respiratory tract symptoms were cough, shortness of breath, and wheeze. Scores were added to give daily upper and lower respiratory tract scores, respectively. A nasal aspirate sample was collected from the child if the upper respiratory tract score was ≥1. The institutes' ethical committee approved the study, project code B04-14.

### 2.1. Sampling

Nasal aspirate samples were collected from children suffering from common cold confirmed diagnosis by the pediatrician based on their symptoms; the sampling method is noninvasive and user-friendly and allows sampling without causing major discomfort. Nasal aspirates were suspended (1 : 10) in virus transport medium and immediately transported to the laboratory on ice, for further processing. Samples were collected within 48 hours of developing respiratory symptoms in all children selected to participate in this study. Two weeks later, control samples were collected from the same children after recovery from infection. A total of 28 samples were collected for this study (symptomatic = 14 and asymptomatic = 14).

### 2.2. Viral Detection

An immunofluorescence kit from Light diagnostics®, Merck Millipore, IFA panel for detection and identification of respiratory viruses, namely, RSV, influenza A, influenza B, adenovirus, and parainfluenza 1, 2, and 3 was used to confirm the virus infection and to detect the causative pathogen. At least 2 cells proved positive for the pathogen. Detection of other viruses was made to prevent selecting samples with coinfection from other viruses.

### 2.3. Differential Cell Counts

Nasal cells obtained from NAs were counted by flow cytometry (FACS Calibur cytometer, Becton Dickinson). All measurements were performed according to the manufacturer's instructions. Cells were suspended at the concentration of 50,000 cells/ml in FACS solution, and they were analyzed by the size and granularity.

### 2.4. HNP-1–4 Measurements

*α*-Defensins were measured in nasal aspirates using specific ELISA kits purchased from Cloud-Clone Corp (alpha-defensins-1 and -3) and MyBioSource (MBS) (alpha-defensins-2 and -4). ELISAs were performed according to the manufacturer's protocols. A tracer antibody conjugated to biotin for capture and streptavidin-peroxidase conjugate to reveal the reaction using the substrate tetramethylbenzidine (TMB) was used. The absorbance was measured at 450 nm on an iMark microplate reader (Bio-Rad). The HNP's concentrations were calculated from each standard curve. The lower limit of detection for HNP-1, HNP-2, HNP-3, and HNP-4 were 0.31 ng/ml, 100 pg/ml, 0.46 ng/ml, and 31.2 pg/ml, respectively.

### 2.5. Statistical Methods

Descriptive statistics were expressed as media (interquartile range); nonparametric analysis was used as two samples. The Mann–Whitney test was used for comparison of defensins values between the groups. The Spearman test was used to evaluate correlations between defensins and cellularity; cell counts were log 10-transformed to facilitate data analysis. Analysis and graphs were calculated using STATA statistical software, version 13 (StataCorp LP, College Station, Texas). In all analysis, *p* < 0.05 was considered statistically significant.

## 3. Results

Between June 2012 and May 2015, 14 children (4.7%) were diagnosed natural adenovirus infection from 280 children (12 females (mean (age): 7.89 (6–12 years))). None of them were taking any medication and clinical symptoms ranged from mild to moderate (none of the children required hospitalization), Table 1. NAs were obtained from all the children during the acute respiratory infection and when asymptomatic.

### 3.1. Measurements of HNP-1–4 in Nasal Secretions

**Figure 1:**
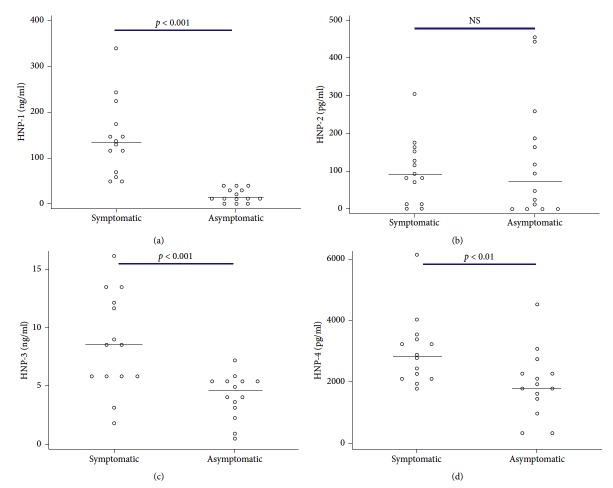
Levels of HNP-1–4 in nasal aspirates from children with naturally occurring adenovirus infection (symptomatic) and when well (asymptomatic). HNP-1–4 were measured by ELISA in nasal aspirates. Statistical analysis and Mann–Whitney *U* test were perfomed.

Measurements of defensins showed increased levels of HNP-1, -3, and -4 in nasal aspirates from children with naturally occurring acute adenovirus respiratory infections as compared with samples from the same children when they had been asymptomatic. Results are expressed as median (range): HNP-1: 125.6 ng/ml (44.4–341) versus 12.9 ng/ml (11.4–22.9) (*p* < 0.001); HNP-3: 8.5 ng/ml (5.8–12.3) versus 4.7 ng/ml (3.2–5.3) (*p* < 0.001); and HNP-4: 2801 pg/ml (2099–3452) versus 1813 pg/ml (1383–2333) (*p* < 0.001), Figures 1, 1, and 1. In contrast, no changes in the levels of HNP-2 had statistical significance of 90 ng/ml (17–153) versus 73 ng/ml (0–187) (*p*=0.9), Figure 1.

### 3.2. Differential Cell Counts

The total cell count measurements were several folds higher in the symptomatic state when compared with the asymptomatic state; median (range) values were 500 (300–1040) cells/mL^−1^ and 48 (32–66) cells/mL^−1^, respectively. All inflammatory cells measured were found to be increased in the symptomatic group (neutrophils, 200-fold; macrophages, 100-fold; and lymphocytes, 150-fold; Table 2) compared with the asymptomatic group. However, eosinophils were present in only few samples, and there was no significant difference (Table 2).

**Figure 2:**
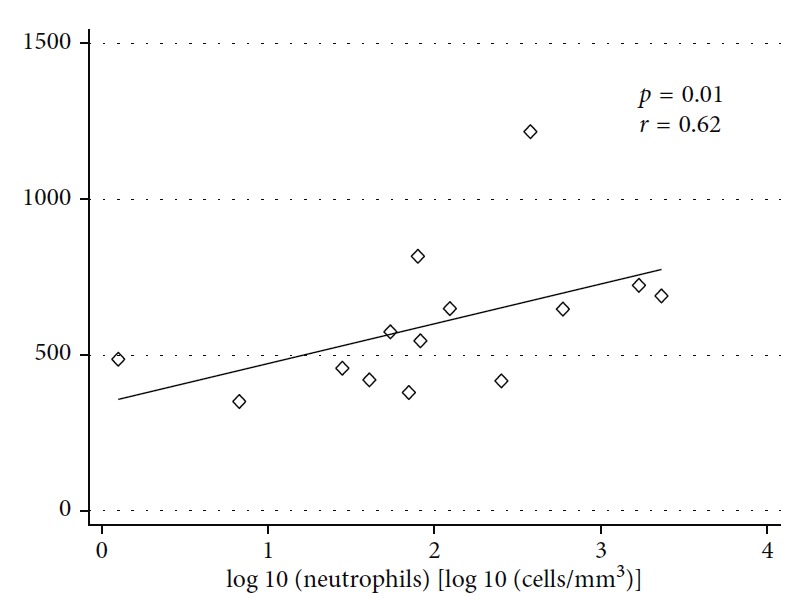
Correlation between HNP-4 and neutrophils in nasal aspirate samples from children with naturally occurring adenovirus infection. Statistical analysis was performed using Spearman's test.

There was a significant positive correlation between HNP-4 and neutrophil numbers in the adenoviral-infected nasal aspirate samples (*p*=0.01; *r*=0.62), Figure 2. In contrast, there was no correlation between *α*-neutrophil numbers and any of the other defensins (HNP-1: *p*=0.08, *r*=−0.48; HNP-2: *p*=0.26, *r*=−0.32; and HNP-3: *p*=0.85, *r*=0.05).

### 3.3. Correlation between HNPs and Symptom Score

To investigate the relationship between HNPs and symptom score, we sought Spearman's rank correlation between the levels of HNP-1, -2, -3, and -4 with symptom score. There was not a significant correlation between HNPs and symptom score (data not shown).

## 4. Discussion

The present study has shown that adenovirus-infected children release high concentrations of HNP-1, -3, and -4. In parallel, we observed increase in neutrophils which is a major source of HNPs. There was no difference in the concentrations of HNP-2. These findings suggest that HNP-1, -3, and -4 are involved in the pathogenesis of adenovirus.

Adenoviruses are nonenveloped viruses whose structure consists of an icosahedral capsid with homotrimeric core and linear nonsegmented double-stranded DNA genome. Innate immunity of the host prevents the virus from entering the cell by directly binding to the virus or its components. However, adenovirus adapts to the microenvironment by degrading some of its structural components and counters innate defense by early proteins, E1A, E1B, E3, and E4, and two noncoding RNAs, VA-I and VA-II. Adenovirus replication then initiates when the viral vertices bind to the host cell surface receptors (CAR or complement protein CD46), and internalization occurs by endocytosis. Eventually, penton attaches to integrins causing vesicle membrane breakage and releasing the viral DNA into the host cell cytoplasm; it migrates to the host nucleus and hijacks the host replication mechanism, using it to produce viral DNA and proteins. Then, infected host cell lysis occurs to release progeny virions.

In the present study, we have shown that adenovirus infection induces the release of the HNP-1, -3, and -4, into the upper airways of 6–12-year-old children. Previous studies have investigated the effect of *α*-defensins on adenovirus. In 2001, Bastian and Schäfer demonstrated that HNP-1, but not HBD-2, neutralizes adenovirus (AdV5) infection in vitro. Indeed, HNP-1 inhibited the adenoviral infection of 293 cells in a concentration range of 2 to 50 mg/ml [17]. In contrast, human beta-defensin- (HBD-) 2 did not reduce infection of 293 cells by AdV5 at doses up to 50 mg/ml [18]. In 2010, Smith et al. showed that not only HNP-1 but also HD-5 inhibited adenoviral infection (A, B1, B2, and C) of A549 airway epithelial cells [19]. Increased HNPs have been found in experimental rhinovirus in 15 asthma patients as compared with 10 normal controls [14]. In this last study, however, the ELISA did not discriminate among the different HNP-1–3 and did not detect HNP-4. In the present study, the use of highly specific ELISAs allowed demonstrating that HNP-1 was released in higher concentrations followed by HNP-3 and HNP-4 in adenovirus-infected children. There was no significant difference in HNP-2 concentrations between the samples. To our knowledge, this study is the first to report the individual concentrations of the 4 HNPs in adenovirus infections. The mechanisms by which HNP-1–4 may inhibit virus infection are not fully understood. A mechanism for selective binding of defensins to nonenveloped viral capsids has been proposed. Fascinating experiments from Nguygen et al. revealed that HD-5 blocked the uncoating mechanism of the virus by stabilizing the capsid in BrdU-labeled HAdV-5 [20]. Single alanine mutagenesis assay revealed HNP-1 and HD-5 require hydrophobic and charged residues for antiadenoviral activity. HNP-1 had more than 2-fold effects of IC50, and when W26 and F28 were substituted by alanine, the mutations were deleterious, which aligned with Y27 and L29 of HD-5 in linear sequence. When these mutations were reversed using nonnatural amino acids, the antiadenoviral activity was restored. These aromatic residues are located on the opposite of beta sheet and stabilize the hydrophobic core [21]. Based on this finding, one could predict HNP-4 may also require hydrophobicity for antiadenovirus activity as F26 and Y28 align linearly with HNP-1–3 and HD-5 [21]. Interestingly, HNP-4 has F26 (aromatic; nonpolar) as F28 in HNP-1–3 and Y28 (aromatic; polar) as HD-5 with Y27 which suggest it may have also antiadenoviral activity. Evidence for an in vivo role of *α*-defensin as antiviral agents in adenovirus infection derives from a study conducted by Gounder et al. showed that a mouse model deficient in activated *α*-defensins in the small intestine is more susceptible to disease upon oral viral infection [22]. Alpha-defensins have also been found to inhibit several enveloped viruses by binding to glycoproteins especially glycoprotein 120 in HIV-1 infection reducing the number of viruses that enter the cell. HNP-4 and HD-6 inhibit HSV-1 and HSV-2 by binding to heparin sulphate, blocking the receptor binding [23, 24].

Neutrophils are known as the major cellular source of HNPs. In our study, neutrophil recruitment was a feature of adenovirus-infected children. Indeed, 200-fold increase in neutrophil numbers was observed during the symptomatic period suggesting these cells migrate into the virus inflammatory site and release HNPs locally. In support of this observation, we found a significant correlation between HNP-4 and neutrophil numbers in the present study.

Interestingly, previous studies have demonstrated that HNPs induce the production neutrophil attractant interleukin (IL)-8 from monocytes and both airway and intestinal epithelial cells in vitro [25, 26]. We and other groups demonstrated that children infected with respiratory viruses including adenovirus induce the release of IL-8 in the upper respiratory airways [27, 28]. Thus, IL-8 production during the adenovirus infection process may recruit neutrophils which in turn release HNPs. These defensins may then stimulate further IL-8 production amplifying neutrophil migration to protect the airways from infection. The lack of correlations between HNPs and score symptoms in the present study raises some doubts about their role in the airways. However, we have previously shown that such correlations are sometimes difficult to find in clinical studies [29] where many variables may affect the strength of a relationship between two biologic measurements as other antivirus mediators are released including interferons [29].

Our study has some potential limitations. First, we did not investigate any of the seven different adenovirus subtypes (A to G). However, subtypes B and C most frequently cause mild respiratory infections, and it is likely that these two adenoviruses subtypes could have infected children participating in this study. And secondly, adenovirus causes more severe symptoms in children below 3 years of age [28] Volunteers participating in this study were 6–12 years, and it is likely that the inflammatory changes in their airways may not mirror those observed in younger children.

In summary, we have shown that children suffering from adenovirus-induced naturally occurring common cold release high concentrations of the HNP-1, -3, and -4, but not HNP-2, in the upper airways. Interestingly, a parallel increased number of neutrophils were observed, suggesting these cells may be a potential source of the HNPs during the adenovirus infection process. The exact mechanism responsible for the increase in HNP levels remains to be elucidated.

## Figures and Tables

**Table 1:** Clinical symptoms presented by AdV-positive children.

Characteristic	HAdV symptomatic (*N*=14)
*Clinical symptoms*	*N* (%)
Blocked nose	13 (92.85)
Throat redness	14 (100)
Fatigue	10 (71.42)
Rhinorrhea	13 (92.8)
Fever	6 (42.85)
Chillness	5 (35.71)
Cough	14 (100)
Headache	7 (50)
Chest pain	5 (35.71)

**Table 2:** Differential and total cell counts in nasal aspirate fluids from symptomatic and asymptomatic.

Cells	Asymptomatic	Symptomatic
Neutrophils	0 (0–16)	52.0 (20.3–79.3)
Macrophages	0 (0–3.0)	24.2 (12.70–44.4)
Lymphocytes	0 (0–0)	7.2 (1.2–23.9)
Eosinophils	0 (0–2.0)	1.9 (0–4.6)
Epithelial	28 (50–34)	4.3 (2.3–15.6)
Total	20 (1–50)	330 (100–4000)

Data represented as median (range) × 10^3^ (symptomatic (*n*=14); asymptomatic (*n*=14)).

## Abbreviations

HNP: Human neutrophil peptide
ELISA: Enzyme-linked immunosorbent assay
AdV: Adenovirus
HPV: Human papillomavirus
HBD: Human beta-defensins
HD: Human defensin
MMP: Matrix metalloproteinase
W: Tryptophan
F: Phenylalanine
Y: Tyrosine
CAR: Coxsackievirus and adenovirus receptor
UTI: Upper respiratory tract infections
TMB: Tetramethylbenzidine.
